# Polymorphisms of *ABCG2* and *SLC22A12* Genes Associated with Gout Risk in Vietnamese Population

**DOI:** 10.3390/medicina55010008

**Published:** 2019-01-07

**Authors:** Nguyen Thuy Duong, Nguyen Thy Ngoc, Nguyen Tran Minh Thang, Bach Thi Hoai Phuong, Nguyen Thanh Nga, Nguyen Doan Tinh, Do Hai Quynh, Nguyen Dang Ton, Nong Van Hai

**Affiliations:** 1 Institute of Genome Research, Vietnam Academy of Science and Technology, 18 Hoang Quoc Viet, Cau Giay, Hanoi 100000, Vietnam; nguyen-thy.ngoc@usth.edu.vn (N.T.N.); nguyentranminhthang@pnt.edu.vn (N.T.M.T.); bachthihoaiphuong93@gmail.com (B.T.H.P.); gemininga92@gmail.com (N.T.N.); dtnguyen@igr.ac.vn (N.D.T.); quynhdohai@gmail.com (D.H.Q.); nguyendoantinha5@gmail.com (N.D.T.); vhnong@igr.ac.vn (N.V.H.); 2 Graduate University of Science and Technology, Vietnam Academy of Science and Technology, 18 Hoang Quoc Viet, Cau Giay, Hanoi 100000, Vietnam; 3 University of Science and Technology of Hanoi, Vietnam Academy of Science and Technology, 18 Hoang Quoc Viet, Cau Giay, Hanoi 100000, Vietnam; 4 Pham Ngoc Thach University of Medicine, 86/2 Thanh Thai, District 10, Ho Chi Minh City 700000, Vietnam

**Keywords:** gout, polymorphism, Vietnamese, *ABCG2*, *SLC22A12*

## Abstract

*Background and objective:* Gout is a common form of inflammatory arthritis caused by the crystallization of uric acid. Previous studies have demonstrated that the genetic predisposition of gout varies in different ethnic populations. However the association study of genetic variants with gout remains unknown in the Vietnamese population. Our study aimed to assess the relationship between polymorphisms in *ABCG2* and *SLC22A12* and gout susceptibility in Vietnamese. *Materials and methods:* Genomic DNA was extracted from blood of a total of 170 patients with gout and 351 healthy controls. We genotyped single nucleotide polymorphisms (SNPs): rs72552713, rs12505410 of the *ABCG2* gene and rs11231825, rs7932775 of the *SLC22A12* gene using polymerase chain reaction–restriction fragment length polymorphism (PCR–RFLP) and then confirmed 10% of randomly selected subjects by Sanger sequencing. *Results:* Three SNPs (rs72552713 and rs12505410 and rs11231825) were in accordance with Hardy–Weinberg Equilibrium (HWE) (*p* > 0.05) while rs7932775 was not (*p* < 0.05). For rs72552713, CT genotype was significantly different between gout patient and control groups (*p* < 0.001) and the T allele was associated with an increased risk of gout (OR = 21.19; 95% CI: 3.00–918.96; *p* < 0.001). Serum uric acid and hyperuricemia differed significantly between CC and CT genotype groups (*p* = 0.004 and 0.008, respectively). For rs11231825, a protective effect against gout risk was identified in the presence of the C allele when compared with the T allele (OR = 0.712; 95% CI: 0.526–0.964 *p* = 0.0302). In contrast, no significant difference of allele frequencies between gout patients and controls was detected for rs12505410 (*p* > 0.05). However, significant differences in serum uric acid and systolic blood pressure were obtained among gout patients. *Conclusion:* Our results suggest that *ABCG2* rs72552713 and *SLC22A12* rs11231825 are likely associated with gout in the Vietnamese population in which T allele may be a risk factor for gout susceptibility.

## 1. Introduction

Gout is a common form of inflammatory arthritis resulting from high levels of serum uric acid (SUA), which can form needle-like crystals in joints. Patients with gout experience substantial pain, redness, warmth and swelling. Gout was reported with an average prevalence of 0.1–10% in the world [1]. In Vietnam, according to the Community Oriented Program for the Control of Rheumatic Diseases (COPCORD) gout prevalence is about 0.14% in Hanoi, the capital city with a population size of about 8 million [2]. With this prevalence level, for the whole country of about 96 million people, the number of gout patients might be tens of thousands. In addition, since the development of gout is not only triggered by environmental factors but also by genetic variants [1], study on the association of genetic variants with gout risk in Vietnamese population is of great interest. Over the last two decades, genome-wide association studies and meta-analyses have identified several genes associated with gout susceptibility, including ATP-binding cassette subfamily G member 2 (*ABCG2*) and solute carrier family 22 member 12 (*SLC22A12*) [3,4].

*ABCG2*, located on chromosome 4q22.1, contains 16 exons spanning over 66 kb. The gene encodes a membrane transporter belonging to the ATP-binding cassette superfamily of membrane transporters, which participates in the trafficking of biomolecules across cell membranes. The ABCG2 protein was first reported to be a xenobiotic transporter that plays a role in the multidrug resistance phenotype of a specific human breast cancer [5]. It is also a high-capacity urate exporter for uric acid excretion in the kidney, liver, and gut [6]. Q141K mutation in the *ABCG2* gene encoded by the common SNP rs2231142 has been shown to cause a 53% reduction in urate transport rate [7]. Patients with dysfunctional variants in *ABCG2* were identified to present symptoms onset 6.5 years earlier than those with the wild-type variant, indicating genetic effects of these variants on gout [8]. Furthermore, several *ABCG2* variants have shown to be associated with gout risk and high SUA in different populations. For examples, rs2231142 is associated with increased SUA levels in American, Dutch, European, Japanese, Chinese, Croatian populations [9,10,11,12,13] while rs2199936 is associated with increased SUA levels and gout risk in Croatian American, Dutch and Icelandic populations [13,14].

*SLC22A12*, encoding urate transporter-1 (URAT1), consists of 10 exons spanning about 11 kb. URAT1 is a urate-anion exchanger that is expressed mainly in the kidney and localized to epithelial cells of the proximal tubule in the renal cortex [15]. Mutations in the *SLC22A12* gene were identified in patients with renal hypouricemia, causing reduced uric acid levels in the blood [16,17]. Although functional consequences of *SLC22A12* polymorphisms have not been fully characterized, several *SLC22A12* variants, including rs475688, rs7932775, rs382507, rs57606, rs7932775 rs11231825 and rs11602903, have been linked to elevated SUA and gout susceptibility [18,19].

While the role of genetic variants of *ABCG2* and *SLC22A12* in the gout pathogenesis has been reported, substantial inconsistencies and certain discrepancies in multiple populations of European and Asian ancestries exist due to the effect of various genes and different genetic backgrounds. It is unclear whether these genes have the same effect in the Vietnamese population, which has different genetic and socio-economic backgrounds. To understand of the relationship between these genes and gout we conducted a case-control association study of *ABCG2* rs72552713, rs12505410 and *SLC22A12* rs11231825, rs7932775 in the Vietnamese population.

## 2. Materials and Methods

### 2.1. Study Subjects

A total of 521 subjects including 170 male patients with gout and 351 healthy controls were enrolled at the Nguyen Trai Hospital, Vietnam during 2016–2017. Gout patients were diagnosed in accordance with the criteria of the American College of Rheumatology [20]. Controls were males, and age-matched healthy individuals, recruited randomly for annual health check with no family history of diabetes or gout. All subjects gave written informed consent before blood collection. Clinical data and laboratory parameters for all subjects were recorded by rheumatologists, including serum levels of GLU (glucose), SUA (serum uric acid), TG (triglyceride), BUN (urea nitrogen), ALT (alanine aminotransferase), AST (aspartate transaminase), CREA (creatinine), HDL-C (high density lipoprotein-cholesterol), LDL-C (low density lipoprotein-cholesterol), CRP (C-reactive protein), WBC (white blood cell count) and age, height, weight and blood pressures. Body mass index was calculated based on the body weight in kg over height square in meter (kg/m^2^). Hyperuricaemia was defined by an abnormally elevated blood level of uric acid, commonly higher than 7 mg/dL in men and 6 mg/dL in women. The study was approved by the Institutional Review Board of the Institute of Genome Research, Vietnam Academy of Science and Technology (No. 1-2017/NCHG-HDDD) and all experimental protocols on human subjects were in accordance with Helsinki Declaration of 1975, as revised in 2008. 

### 2.2. SNP Genotyping

Peripheral blood samples were collected for genomic DNA extraction, using a DNeasy blood and tissue kit (Qiagen, Hilden, Germany) according to the manufacturer’s protocols. To genotype SNPs of *ABCG2* (rs72552713 and rs12505410) and *SLC22A12* (rs11231825 and rs7932775), polymerase chain reaction–restriction fragment length polymorphism (PCR–RFLP) was performed using specific primers. The primers designed using Primer 3 software were listed in Table 1. PCR products were digested with restriction enzymes *Rsa*I, *Nsi*I, *Bcl*I and *Eco130*I for rs72552713, rs12505410, rs11231825 and rs7932775, respectively. To verify the results obtained from PCR-RFLP, 10% of subjects were randomly selected for direct sequencing using ABI PRISM 3500 genetic analyzer (Applied Bio-systems, Carlsbad, CA, USA). The obtained sequences were compared to *ABCG2* and *SLC22A12* sequences published in GenBank with respective accession numbers NM_004827 and NM_153378. The results showed that there was 100% concordance of observed genotypes obtained from PCR-RFLP and Sanger sequencing in the samples checked.

### 2.3. Statistical Analyses

We used SPSS version 23 (IBM, New York, NY, USA), Microsoft Excel (Microsoft Corp., Washington, DC, USA) for statistical analyses. Chi-squared test (𝜒^2^) was used to test whether allele distribution of each SNP follows Hardy–Weinberg Equilibrium (HWE). To test three models (additive, dominant, recessive) for associations of the studied SNPs with categorical variables (gout and hyperuricemia), Fisher’s exact test was used for SNPs with expected sample sizes less than 5 and Chi-squared test for those with larger expected sample sizes. The normality of data distribution was checked by using the Shapiro–Wilk test. Associations of each SNP with other quantitative variables were assessed using the Mann–Whitney U or Kruskal–Wallis tests since the data distributions were not normal. The odds ratios and 95% confidence intervals were calculated based on the formula described by Szumilas in 2010 [21]. All of the statistical tests were two-sided. *p* < 0.05 was considered statistically significant. 

## 3. Results

### 3.1. Comparison of Clinical Characteristics between Gout Patient and Control Groups

A total of 521 subjects including 170 gout patients and 351 controls with demographic and clinical data described in Table 2 were recruited for this study. The most striking differences between gout patient and control groups were SUA and hyperuricaemia with p values less than 0.001. The controls had lower weight, BMI, CRP levels, LDL-C levels and TG levels than the gout patients (*p* < 0.05). In contrast, there were no significant differences in age, height, ALT levels, AST levels, BUN, CREA levels, diastolic BP, systolic BP, GLU levels, HDL-C levels and WBC between the two groups (*p* > 0.05) (Table 2).

### 3.2. Genetic Analysis of ABCG2 and SLC22A12 SNPs

Polymorphisms of *ABCG2 (*rs72552713 and rs12505410) and *SLC22A12* (rs11231825 and rs7932775) were genotyped for all studied subjects. Among the four SNPs, the genotype distributions of rs72552713, rs12505410 and rs11231825 were in accordance with HWE (*p* > 0.05) (Table 3) while rs7932775 was not (*p* < 0.05). The minor allele frequency (MAF) of *ABCG2* rs72552713 in the gout group was much higher than that in the control group, implying a correlation between this risk allele and disease susceptibility. Other SNPs did not show clear discrepancies of MAFs when compared between the two groups. 

Statistical analyses were performed on three models of minor alleles (additive, dominant and recessive). For *ABCG2* rs72552713, the association of genotypes with gout was tested in only the dominant model because TT genotype was not present in the studied population. A significant difference of CT genotype between gout patient and control groups was detected when compared with CC genotype (*p* < 0.001) and T allele was associated with an increased risk of gout (OR = 21.19, 95% CI: 3.00-918.96, *p* < 0.001) (Table 4). In contrast, no significant difference in allele frequencies between gout patient and control groups was found for *ABCG2* rs12505410 in all three tested models (*p* > 0.05). For *SLC22A12* rs11231825, a significant difference of genotypes was obtained in the additive model (*p* = 0.021). Both gout patient and control groups had high frequencies of TT genotype with 58.2% and 52.1%, respectively. The frequency of CC genotype differed significantly between the two groups and was associated with a decreased risk of gout (OR = 0.272, 95% CI: 0.103–0.717, *p* = 0.005) compared to the TT genotype. No significant difference of TC genotype between the two groups was obtained in comparison to the TT genotype (*p* = 0.631). Similarly, in the recessive model the CC genotype was linked to a reduced gout risk when compared to the combination of TT + TC genotype group (OR = 0.283, 95% CI: 0.108–0.736, *p* = 0.006); the C allele lowered gout susceptibility (OR = 0.712, 95% CI: 0.526–0.964, *p* = 0.0302) in comparison to the T allele. We did not conduct genetic association tests for *SLC22A12* rs7932775, because its genotype distribution among the whole studied population did not follow HWE (*p* = 0.003).

### 3.3. Association of SNPs Genotypes and Clinical Characteristics among the Studied Population

For *ABCG2* rs72552713, SUA levels were higher in CT genotype than in CC genotype group in a dominant model, suggesting T allele was a risk factor for increased SUA levels (*p* = 0.004) (Table 5). There was also a significantly higher frequency of hyperuricemia in the CT genotype than in the CC genotype group (*p* = 0.008). For *SLC22A12* rs11231825, CC genotype had lower levels of SUA when compared to the combination of TT + TC genotype group in a recessive model (*p* = 0.018) (Table 5). In contrast, *ABCG2* rs12505410 genotype groups were not correlated with both SUA levels and hyperuricemia (*p* > 0.05). 

### 3.4. Analysis of Genotype and Phenotype Correlation in ABCG2 rs12505410 among Gout Patients

Significant differences in SUA levels and systolic BP were obtained in the recessive model (TT + TG/GG) (*p* = 0.003 and *p* = 0.045, respectively) (Table 6). Moreover, SUA levels were significantly different when gout patients were divided into TT, TG and GG genotype groups (*p* = 0.011). However, CREA, CRP and diastolic BP did not differ significantly among these groups (*p* > 0.05).

## 4. Discussion

Gout is often associated with many risk factors like high protein, alcohol, drugs, obesity, hypertension and hyperuricaemia [22]. Consistent with previous studies, we found associations of SUA, hyperuricaemia, weight, BMI, LDL-C, and TG with gout [22,23]. Moreover, we found higher levels of CRP in gout patients than in the control group. This finding is likely due to the fact that most gout patients were experiencing acute inflammation at the time of giving their blood samples. 

Genome-wide association studies and meta-analysis have identified several genes associated with gout susceptibility [3,4,24,25]. Among these, *ABCG2* and *SLC22A12* genes are known risk factors for gout in different populations. The prevalence of gout is increasing in Vietnam as the population becomes more overweight and obese [26,27]. However, to our knowledge, no study has assessed the association between these two genes and gout susceptibility in the Vietnamese population. Common genetic variants of the *ABCG2* gene, such as C376T, have been strongly associated with gout risk in various populations [24,28,29]. The C376T mutation changes a glutamine to a stop codon Q126X, referred to as rs72552713, which is a stop-gained variant and causes the loss of ABCG2 protein function. In accordance with previous reports, we found a significant association between *ABCG2* rs72552713 and an elevated risk of gout in the Vietnamese population [11,30]. Furthermore, the levels of SUA were higher in CT heterozygous carriers than in CC homozygote carriers [31]. This variant was also correlated with hyperuricemia and high SUA levels [31]. However, our finding is different from a study of gout in the Han Taiwanese population where rs72552713 was not associated with SUA levels [32]. Given the multifactorial pathogenesis of gout, this inconsistency might be due to genetic backgrounds of different populations, different socio-economic status and lifestyle. Additionally, we assessed another rare variant of the *ABCG2* gene, rs12505410. This SNP was not only found to be associated with gout susceptibility and high SUA levels in the Han Chinese population [33,34] but also with cancer susceptibility [35,36]. In contrast, we did not find any correlation between rs12505410 and gout risk but obtained significant associations between the SNP and SUA levels and systolic BP among gout patients when divided into different genotypes. Future studies should aim to determine the impact of ABCG2 rs12505410 on systolic blood pressure in larger/genetically different populations as well as the mechanism of this effect.

The *SLC22A12* gene encodes urate transporter 1 (URAT1), which is the main transporter involved in uric acid reabsorption in the luminal membrane. Different loss-of-function mutations or variants in the *SLC22A12* gene have been identified in patients with renal hypouricemia (lack of serum uric acid) [15,16,17,37,38]. On the other hand, *SLC22A12* rs11231825 has been shown to be associated with reduced renal urate excretion and hyperuricemia in German and Han Chinese populations [19,39]. In the present study, rs11231825 was correlated with elevated SUA levels (*p* = 0.018) but not with hyperuricemia (*p* = 0.076). The allele associated with an increased risk of hyperuricemia for both the German and the Han Chinese populations was the “T” allele. In Vietnamese population, under a recessive model, the SUA levels of the combination of TT + TC genotype group were higher than that of CC group (*p* = 0.018). Although hyperuricemia was not significantly different between (TT + TC) and CC genotype groups (*p* = 0.076) carriers of one or two “T” alleles had a higher risk of hyperuricemia when compared to CC carriers (63.1% and 48.7%, respectively). Similar to our results, Tabara and co-workers showed that carriers of one or two “T” alleles had higher SUA levels than CC carriers in Japanese population (TT/TC/CC: 5.3 ± 1.4/5.0 ± 1.4/4.5 ± 1.6 mg/dL, p = 7.6 × 10^-20^) [16]. Furthermore, similar to our study in both additive and recessive models, Torres et al. showed that carriers of one or two minor alleles “T” increased risk of gout in Spanish population (OR = 1.631, 95% CI: 1.038–2.561, *p* = 0.0326) [40]. Interestingly, to our knowledge, our result provides an evidence of the association between *SLC22A12* rs11231825 with gout susceptibility in an Asian population. 

### Limitations of the Study

Our study had several limitations that should be taken in account. First, the sample size was not sufficient to confirm potential association between two of the SNPs (rs7932775 and rs12505410) and gout risk in the Vietnamese population. Second, all gout patients and healthy controls were recruited from the same hospital. Therefore selection bias could have contributed to the results. In spite of these restrictions, our study was the first to reveal associations between SNPs (rs72552713 in *ABCG2*, and rs11231825 in *SLC22A12*) and gout among Vietnamese. These SNPs should be considered when constructing predictive model for early detection of gout in Vietnamese population. While several studies have reported the relationship between *ABCG2* and *SLC22A12* variants and gout in various ethnic populations, to our best knowledge, the current study was the first one to investigate the genotype distributions and effects of these SNPs on the Vietnamese population.

## 5. Conclusions

In addition to previous reports in different populations, our results provide more evidence on the association of *ABCG2* rs72552713 and *SLC22A12* rs11231825 with gout and serum uric acid in the Vietnamese population. Additionally, we found that *ABCG2* rs12505410 had signification differences of genotypes in serum uric acid levels and systolic blood pressure among gout patients. Further investigation should be implemented to obtain more information on the association of *ABCG2* and *SLC22A12* SNPs or other different genetic variants with gout. 

## Figures and Tables

**Table 1:** List of primers used for polymerase chain reaction–restriction fragment length polymorphism (PCR–RFLP) amplification and sequencing.

PCR Primer Sequence			
**Gene/SNP**	**Forward Primer (5′-3′)**	**Reverse Primer (5′-3′)**	**Fragment Size (bp)**
*ABCG2*/rs72552713	AGCTGCAAGGAAAGATCCAA	GGGTAAGTGCTTTGGCTGAT	166
*ABCG2*/rs12505410	CCCTTGGCACCTTAAATGAA	ATAGGTGGCTGGCCCTATTT	308
*SLC22A12*/rs11231825	CCCTAGAGGTCACCAGACCA	ACTGGGCCATGGGCTTCTGATC	168
*SLC22A12*/rs7932775	GCCTGAAAGTCAGGGACAAG	ACCACACAAGAGGGAGATGC	325
**Gene/SNP**	**Sequencing Primer (5′-3′)**		
*ABCG2*/rs72552713	AGCTGCAAGGAAAGATCCAA		
*ABCG2*/rs12505410	CCCTTGGCACCTTAAATGAA		
*SLC22A12*/rs11231825	CCCTAGAGGTCACCAGACCA		
*SLC22A12*/rs7932775	GCCTGAAAGTCAGGGACAAG		

**Table 2:** Demographic and clinical characteristics of gout patients and controls.

Characteristics	Controls (*n* = 351)	Gout Patients (*n* = 170)	Total (*n* = 521)	*p*-Value
Age	52 (13)	52 (20)	52 (15)	0.469 ^(2)^
Height (cm)	165 (8)	165 (9.3)	165 (9)	0.697 ^(2)^
Weight (kg)	66 (11)	67 (13)	66 (12)	0.026 ^(2)^
BMI (kg/m^2^)	24.11 (4.4)	24.78 (3.83)	24.31 (4.14)	0.038 ^(2)^
SUA (mg/dL)	6.9 (1.98)	9.25 (2.18)	7.61 (2.66)	<0.001 ^(2)^
ALT (U/L)	26.4 (18.8)	26.05 (21.52)	26.2 (18.95)	0.567 ^(2)^
AST (U/L)	23.1 (9.1)	23.1 (11.2)	23.1 (9.95)	0.354 ^(2)^
BUN (mg/dL)	21.4 (6.1)	22.52 (10.07)	21.93 (7.9)	0.053 ^(2)^
CREA (mg/dL)	1.06 (0.17)	1.07 (0.16)	1.06 (0.17)	0.722 ^(2)^
CRP (mg/dL)	1.56 (4.34)	3.53 (6.7)	2.02 (5.24)	<0.001 ^(2)^
Diastolic BP (mmHg)	70 (10)	70 (10)	70 (10)	0.533 ^(2)^
Systolic BP (mmHg)	120 (20)	120 (20)	120 (20)	0.84 ^(2)^
GLU (mg/dL)	101 (13)	103 (15.75)	101 (14)	0.145 ^(2)^
HDL-C (mg/dL)	45.6 (16)	43.75 (19.12)	44.6 (16.6)	0.204 ^(2)^
LDL-C (mg/dL)	120.8 (53.8)	126 (56.65)	122.3 (53.9)	0.04 ^(2)^
Hyperuricemia *	166 (47.3%)	157 (92.4%)	323 (62%)	<0.001 ^(1)^
TG (mg/dL)	169.2 (133.4)	231.2 (164.85)	189.2 (142.35)	0.001 ^(2)^
WBC (per µL)	7300 (2550)	7110 (2477.5)	7260 (2540)	0.778 ^(2)^

ALT (alanine aminotransferase), AST (aspartate transaminase), BMI (body mass index), BUN (blood urea nitrogen), BP (blood pressure), CREA (creatinine), CRP (C-reactive protein), GLU (glucose), HDL-C (high density lipoprotein-cholesterol), LDL-C (low density lipoprotein-cholesterol), SUA (serum uric acid), TG (triglyceride) and WBC (white blood cell count); Data presented as either counts (percentage) * or median (interquartile range); n: number of individuals in group; ^(1)^ *p*-value calculated by Chi-squared test; ^(2)^ *p*-value calculated by Mann–Whitney U test; *p* < 0.05 (in bold) indicates statistical significance.

**Table 3:** General information on the studied single nucleotide polymorphisms (SNPs).

Gene/SNP	Position	Type of Variant	Allele	MAF in Gout Group	HWE in Gout Group (*p*)	MAF in Control Group	HWE in Control Group (*p*)	HWE in all Population (*p*)
*ABCG2*/rs72552713	4:88131805	Stop gained	C/T	0.029	0.925	0.001	1.000	0.97
*ABCG2*/rs12505410	4:88109689	Intron	T/G	0.403	0.374	0.385	1.000	0.708
*SLC22A12*/rs11231825	11:64592802	Synonymous	T/C	0.224	0.304	0.288	0.438	0.914
*SLC22A12*/rs7932775	11:64600390	Missense	C/T	0.418	0.03	0.487	0.074	0.003

Position refers to the GRCh38.p10 assembly; MAF: Minor allele frequency; HWE: Hardy-Weinberg equilibrium was checked by Chi-squared test.

**Table 4:** Associations of *ABCG2* and *SLC22A12* SNPs with gout.

SNP/Gene	Test model	Controls (*n* = 351)	Gout patients (*n* = 170)	OR	95% CI	*p*-Value
*rs72552713*	Dominant					
*(ABCG2)*	CC	350 (99.7%)	160 (94.1%)	1.00		
	CT	1 (0.3%)	10 (5.9%)	21.875	2.77–172.34	<0.001 ^(1)^
	Alleles					
	C	701 (99.86%)	330 (97.06%)	1.00		
	T	1 (0.14%)	10 (2.94%)	21.19	3.00–918.96	<0.001 ^(1)^
*rs12505410*	Additive					0.458 ^(2)^
*(ABCG2)*	TT	133 (37.9%)	66 (38.8%)	1.00		
	TG	167 (47.6%)	73 (42.9%)	0.881	0.588–1.319	0.538 ^(2)^
	GG	51 (14.5%)	31 (18.2%)	1.225	0.717–2.092	0.457 ^(2)^
	Dominant					
	TT	133 (37.9%)	66 (38.8%)	1.00		
	TG + GG	218 (62.1%)	104 (61.2%)	0.961	0.66–1.401	0.837 ^(2)^
	Recessive					
	TT + TG	300 (85.5%)	139 (81.8%)	1.00		
	GG	51 (14.5%)	31 (18.2%)	1.312	0.804–2.141	0.276 ^(2)^
	Alleles					
	T	433 (61.7%)	205 (60.3%)	1.00		
	G	269 (38.3%)	135 (39.7%)	1.06	0.805–1.393	0.684 ^(2)^
*rs11231825*	Additive					0.021 ^(2)^
*(SLC22A12)*	TT	183 (52.1%)	99 (58.2%)	1.00		
	TC	134 (38.2%)	66 (38.8%)	0.91	0.621–1.335	0.631 ^(2)^
	CC	34 (9.7%)	5 (2.9%)	0.272	0.103–0.717	0.005 ^(2)^
	Dominant					
	TT	183 (52.1%)	99 (58.2%)	1.00		
	TC + CC	168 (47.9%)	71 (41.8%)	0.781	0.54–1.131	0.19 ^(2)^
	Recessive					
	TT + TC	317 (90.3%)	165 (97.1%)	1.00		
	CC	34 (9.7%)	5 (2.9%)	0.283	0.108–0.736	0.006 ^(2)^
	Alleles					
	T	500 (71.2%)	264 (77.6%)	1.00		
	C	202 (28.8%)	76 (22.4%)	0.712	0.526–0.964	0.0302 ^(2)^

95% CI: 95% confidence interval of odds ratio; *p*-values calculated by either Fisher’s exact test ^(1)^ or Chi-squared test ^(2)^; *p* < 0.05 (in bold) indicates statistical significance; OR: Odds ratio.

**Table 5:** Association of SNPs with clinical characteristics among the studied population.

SNP/Gene	Test Model	Genotype	Uric Acid		Hyperuricemia	
			Median (Interquartile Range)	*p*-Value ^(1)^	Positive *n* (%)	*p*-Value ^(2)^
*rs72552713*	Dominant			0.004		0.008
*(ABCG2)*		CC	7.57 (2.65)		311 (61%)	
		CT	9.2 (2.28)		11 (100%)	
*rs12505410*	Additive			0.131		0.791
*(ABCG2)*		TT	7.66 (2.77)		126 (63.6%)	
		TG	7.65 (2.67)		147 (61.5%)	
		GG	7.45 (2.47)		50 (59.5%)	
	Dominant			0.105		0.546
		TT	7.66 (2.77)		126 (63.6%)	
		TG + GG	7.58 (2.65)		197 (61%)	
	Recessive			0.092		0.61
		TT + TG	7.65 (2.47)		273 (62.5%)	
		GG	7.45 (2.47)		50 (59.5%)	
*rs11231825*	Additive			0.057		0.178
*(SLC22A12)*		TT	7.6 (2.7)		175 (62.1%)	
		TC	7.7 (2.6)		129 (64.5%)	
		CC	7.0 (2.6)		19 (48.7%)	
	Dominant			0.647		0.975
		TT	7.6 (2.7)		175 (62.1%)	
		TC + CC	7.61 (2.73)		148 (61.9%)	
	Recessive			0.018		0.076
		TT + TC	7.69 (2.66)		304 (63.1%)	
		CC	7.0 (2.6)		19 (48.7%)	

Data presented as median (interquartile range); *n*: number of individuals in group; p-values calculated by either Mann-Whitney U test ^(1)^ or Chi-squared test ^(2)^; *p* < 0.05 (in bold) indicates statistical significance.

**Table 6:** Association of rs12505410 genotypes and clinical characteristics among gout patients.

rs12505410	Genotype			*p*-Value Additive (TT/TG/GG)	*p*-Value Dominant (TT/TG + GG)	*p*-Value Recessive (TT + TG/GG)
	TT (*n* = 65)	TG (*n* = 73)	GG (*n* = 32)			
CREA (mg/dL)	1.04 (0.26)	1.07 (0.17)	1.1 (0.12)	0.444	0.291	0.289
CRP (mg/dL)	3.67 (5.8)	3.45 (6.2)	3.68 (7.9)	0.661	0.992	0.398
Diastolic BP (mmHg)	70 (10)	80 (10)	70 (10)	0.385	0.262	0.749
SUA (mg/dL)	9.54 (2.38)	9.62 (2.12)	8.5 (1.48)	0.011	0.23	0.003
Systolic BP (mmHg)	120 (20)	120 (20)	110 (17.5)	0.064	0.707	0.045

CREA (creatinine), CRP (C-reactive protein), BP (blood pressure), SUA (serum uric acid); Data presented as median (interquartile range); n: number of individuals in group; *p*-values calculated by Mann–Whitney U test; *p* < 0.05 (in bold) indicates statistical significance.

## References

[1] Kuo C.F., Grainge M.J., Zhang W., Doherty M. (2015). Global epidemiology of gout: Prevalence, incidence and risk factors. Nat. Rev. Rheumatol..

[2] Minh Hoa T.T., Darmawan J., Chen S.L., Van Hung N., Thi Nhi C., Ngoc An T. (2003). Prevalence of the rheumatic diseases in urban Vietnam: A WHO-ILAR COPCORD study. J. Rheumatol..

[3] Kottgen A., Albrecht E., Teumer A., Vitart V., Krumsiek J., Hundertmark C., Pistis G., Ruggiero D., O’Seaghdha C.M., Haller T. (2013). Genome-wide association analyses identify 18 new loci associated with serum urate concentrations. Nat. Genet..

[4] Tin A., Woodward O.M., Kao W.H., Liu C.T., Lu X., Nalls M.A., Shriner D., Semmo M., Akylbekova E.L., Wyatt S.B. (2011). Genome-wide association study for serum urate concentrations and gout among African Americans identifies genomic risk loci and a novel urat1 loss-of-function allele. Hum. Mol. Genet..

[5] Doyle L.A., Yang W., Abruzzo L.V., Krogmann T., Gao Y., Rishi A.K., Ross D.D. (1998). A multidrug resistance transporter from human mcf-7 breast cancer cells. Proc. Natl. Acad. Sci. USA.

[6] Saison C., Helias V., Ballif B.A., Peyrard T., Puy H., Miyazaki T., Perrot S., Vayssier-Taussat M., Waldner M., Le Pennec P.Y. (2012). Null alleles of abcg2 encoding the breast cancer resistance protein define the new blood group system junior. Nat. Genet..

[7] Woodward O.M., Kottgen A., Coresh J., Boerwinkle E., Guggino W.B., Kottgen M. (2009). Identification of a urate transporter, abcg2, with a common functional polymorphism causing gout. Proc. Natl. Acad. Sci. USA.

[8] Matsuo H., Ichida K., Takada T., Nakayama A., Nakashima H., Nakamura T., Kawamura Y., Takada Y., Yamamoto K., Inoue H. (2013). Common dysfunctional variants in abcg2 are a major cause of early-onset gout. Sci. Rep..

[9] Dehghan A., Kottgen A., Yang Q., Hwang S.J., Kao W.L., Rivadeneira F., Boerwinkle E., Levy D., Hofman A., Astor B.C. (2008). Association of three genetic loci with uric acid concentration and risk of gout: A genome-wide association study. Lancet.

[10] Kolz M., Johnson T., Sanna S., Teumer A., Vitart V., Perola M., Mangino M., Albrecht E., Wallace C., Farrall M. (2009). Meta-analysis of 28,141 individuals identifies common variants within five new loci that influence uric acid concentrations. PLoS Genet..

[11] Matsuo H., Takada T., Ichida K., Nakamura T., Nakayama A., Ikebuchi Y., Ito K., Kusanagi Y., Chiba T., Tadokoro S. (2009). Common defects of abcg2, a high-capacity urate exporter, cause gout: A function-based genetic analysis in a Japanese population. Sci. Transl. Med..

[12] Wang B., Miao Z., Liu S., Wang J., Zhou S., Han L., Meng D., Wang Y., Li C., Ma X. (2010). Genetic analysis of abcg2 gene c421a polymorphism with gout disease in Chinese Han male population. Hum. Genet..

[13] Karns R., Zhang G., Sun G., Rao Indugula S., Cheng H., Havas-Augustin D., Novokmet N., Rudan D., Durakovic Z., Missoni S. (2012). Genome-wide association of serum uric acid concentration: Replication of sequence variants in an island population of the Adriatic coast of Croatia. Ann. Hum. Genet..

[14] Yang Q., Kottgen A., Dehghan A., Smith A.V., Glazer N.L., Chen M.H., Chasman D.I., Aspelund T., Eiriksdottir G., Harris T.B. (2010). Multiple genetic loci influence serum urate levels and their relationship with gout and cardiovascular disease risk factors. Circ. Cardiovasc. Genet..

[15] Enomoto A., Kimura H., Chairoungdua A., Shigeta Y., Jutabha P., Cha S.H., Hosoyamada M., Takeda M., Sekine T., Igarashi T. (2002). Molecular identification of a renal urate anion exchanger that regulates blood urate levels. Nature.

[16] Tanaka M., Itoh K., Matsushita K., Matsushita K., Wakita N., Adachi M., Nonoguchi H., Kitamura K., Hosoyamada M., Endou H. (2003). Two male siblings with hereditary renal hypouricemia and exercise-induced arf. Am. J. Kidney Dis..

[17] Ichida K., Hosoyamada M., Kamatani N., Kamitsuji S., Hisatome I., Shibasaki T., Hosoya T. (2008). Age and origin of the g774a mutation in slc22a12 causing renal hypouricemia in Japanese. Clin. Genet..

[18] Tu H.P., Chen C.J., Lee C.H., Tovosia S., Ko A.M., Wang S.J., Ou T.T., Lin G.T., Chiang S.L., Chiang H.C. (2010). The slc22a12 gene is associated with gout in Han Chinese and Solomon islanders. Ann. Rheum. Dis..

[19] Li C., Han L., Levin A.M., Song H., Yan S., Wang Y., Wang Y., Meng D., Lv S., Ji Y. (2010). Multiple single nucleotide polymorphisms in the human urate transporter 1 (hurat1) gene are associated with hyperuricaemia in Han Chinese. J. Med. Genet..

[20] Neogi T., Jansen T.L., Dalbeth N., Fransen J., Schumacher H.R., Berendsen D., Brown M., Choi H., Edwards N.L., Janssens H.J. (2015). 2015 gout classification criteria: An American college of rheumatology/European league against rheumatism collaborative initiative. Arthritis Rheumatol..

[21] Szumilas M. (2010). Explaining odds ratios. J. Can. Acad Child. Adolesc. Psychiatry.

[22] Doherty M. (2009). New insights into the epidemiology of gout. Rheumatology (Oxford).

[23] Singh J.A., Reddy S.G., Kundukulam J. (2011). Risk factors for gout and prevention: A systematic review of the literature. Curr. Opin. Rheumatol..

[24] Li C., Li Z., Liu S., Wang C., Han L., Cui L., Zhou J., Zou H., Liu Z., Chen J. (2015). Genome-wide association analysis identifies three new risk loci for gout arthritis in Han Chinese. Nat. Commun..

[25] Matsuo H., Yamamoto K., Nakaoka H., Nakayama A., Sakiyama M., Chiba T., Takahashi A., Nakamura T., Nakashima H., Takada Y. (2016). Genome-wide association study of clinically defined gout identifies multiple risk loci and its association with clinical subtypes. Ann. Rheum. Dis..

[26] Khan N.C., Khoi H.H. (2008). Double burden of malnutrition: The Vietnamese perspective. Asia Pac. J. Clin. Nutr..

[27] Son L.N.T., Kunii D., Hung N.T.K., Sakai T., Yamamoto S. (2005). The metabolic syndrome: Prevalence and risk factors in the urban population of Ho Chi Minh city. Diabetes Res. Clin. Pract..

[28] Kim Y.S., Kim Y., Park G., Kim S.K., Choe J.Y., Park B.L., Kim H.S. (2015). Genetic analysis of abcg2 and slc2a9 gene polymorphisms in gouty arthritis in a Korean population. Korean J. Intern. Med..

[29] Nakayama A., Nakaoka H., Yamamoto K., Sakiyama M., Shaukat A., Toyoda Y., Okada Y., Kamatani Y., Nakamura T., Takada T. (2017). Gwas of clinically defined gout and subtypes identifies multiple susceptibility loci that include urate transporter genes. Ann. Rheum. Dis..

[30] Li R., Miao L., Qin L., Xiang Y., Zhang X., Peng H., Mailamuguli, Sun Y., Yao H. (2015). A meta-analysis of the associations between the q141k and q126x abcg2 gene variants and gout risk. Int. J. Clin. Exp. Pathol..

[31] Nakayama A., Matsuo H., Nakaoka H., Nakamura T., Nakashima H., Takada Y., Oikawa Y., Takada T., Sakiyama M., Shimizu S. (2014). Common dysfunctional variants of abcg2 have stronger impact on hyperuricemia progression than typical environmental risk factors. Sci. Rep..

[32] Cheng S.T., Wu S., Su C.W., Teng M.S., Hsu L.A., Ko Y.L. (2017). Association of abcg2 rs2231142-a allele and serum uric acid levels in male and obese individuals in a Han Taiwanese population. J. Formos Med. Assoc..

[33] Li Z., Zhou Z., Hou X., Lu D., Yuan X., Lu J., Wang C., Han L., Cui L., Liu Z. (2017). Replication of gout/urate concentrations gwas susceptibility loci associated with gout in a Han Chinese population. Sci. Rep..

[34] Yu K.H., Chang P.Y., Chang S.C., Wu-Chou Y.H., Wu L.A., Chen D.P., Lo F.S., Lu J.J. (2017). A comprehensive analysis of the association of common variants of abcg2 with gout. Sci. Rep..

[35] Campa D., Pardini B., Naccarati A., Vodickova L., Novotny J., Forsti A., Hemminki K., Barale R., Vodicka P., Canzian F. (2008). A gene-wide investigation on polymorphisms in the abcg2/brcp transporter and susceptibility to colorectal cancer. Mutat. Res..

[36] Delord M., Rousselot P., Cayuela J.M., Sigaux F., Guilhot J., Preudhomme C., Guilhot F., Loiseau P., Raffoux E., Geromin D. (2013). High imatinib dose overcomes insufficient response associated with abcg2 haplotype in chronic myelogenous leukemia patients. Oncotarget.

[37] Wakida N., Tuyen D.G., Adachi M., Miyoshi T., Nonoguchi H., Oka T., Ueda O., Tazawa M., Kurihara S., Yoneta Y. (2005). Mutations in human urate transporter 1 gene in presecretory reabsorption defect type of familial renal hypouricemia. J. Clin. Endocrinol. Metab..

[38] Iwai N., Mino Y., Hosoyamada M., Tago N., Kokubo Y., Endou H. (2004). A high prevalence of renal hypouricemia caused by inactive slc22a12 in japanese. Kidney Int..

[39] Graessler J., Graessler A., Unger S., Kopprasch S., Tausche A.K., Kuhlisch E., Schroeder H.E. (2006). Association of the human urate transporter 1 with reduced renal uric acid excretion and hyperuricemia in a German Caucasian population. Arthritis Rheum..

[40] Torres R.J., de Miguel E., Bailen R., Banegas J.R., Puig J.G. (2014). Tubular urate transporter gene polymorphisms differentiate patients with gout who have normal and decreased urinary uric acid excretion. J. Rheumatol..

